# Supplementary material for: Splice-Junction-Based Mapping of Alternative Isoforms in the Human Proteome
Source: Cell Rep. Author manuscript; Available in PMC 2020 Jan 15. (PMC6961840; doi:10.1016/j.celrep.2019.11.026)
Supplement: 2 [file NIHMS1546469-supplement-2.zip › DF1/PXD010154/Lung/Lung_41_ST3GAL5_DLSGGIDREGAASPAAADR.pdf]

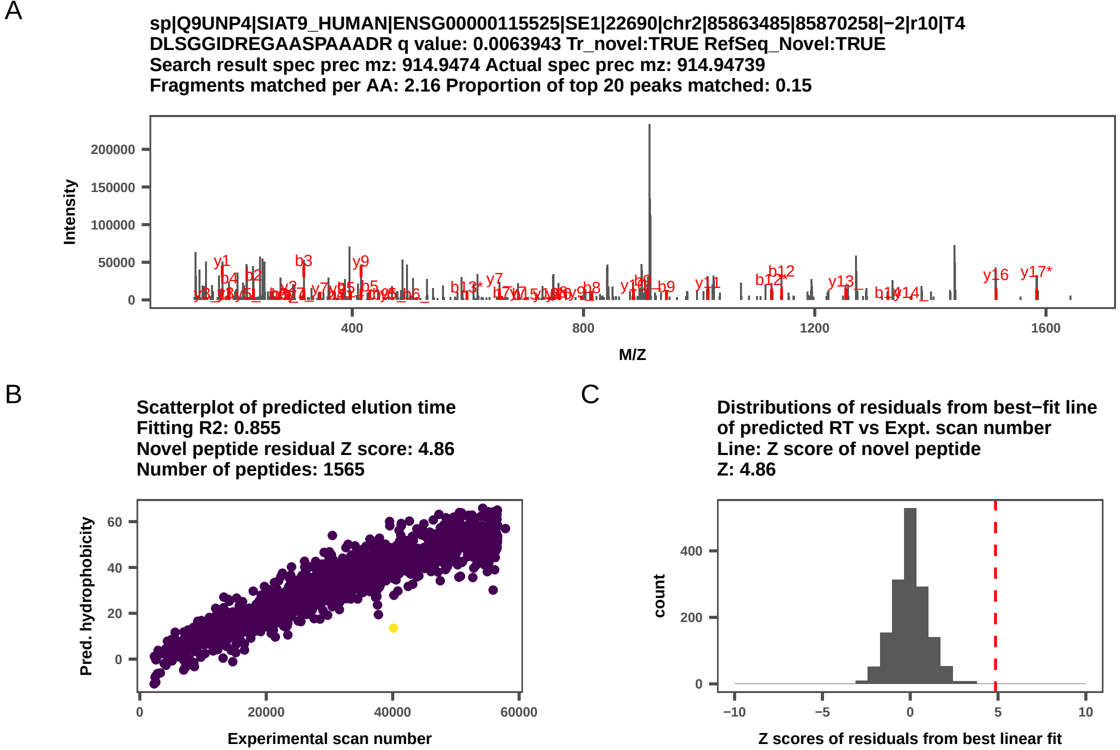
A
sp|Q9UNP4|SIAT9_HUMAN|ENSG00000115525|SE1|22690|chr2|85863485|85870258|−2|r10|T4
DLSGGIDREGAASPAAADR q value: 0.0063943 Tr_novel:TRUE RefSeq_Novel:TRUE
Search result spec prec mz: 914.9474 Actual spec prec mz: 914.94739
Fragments matched per AA: 2.16 Proportion of top 20 peaks matched: 0.15
Intensity
200000
150000
100000
50000
0
y1
b3
y9
b4
b2
y7
b13*
b8
b9
y11
b12
y13
y16
y17*
400
800
1200
1600
M/Z
B
Scatterplot of predicted elution time
Fitting R2: 0.855
Novel peptide residual Z score: 4.86
Number of peptides: 1565
Pred. hydrophobicity
60
40
20
0
0
20000
40000
60000
Experimental scan number
C
Distributions of residuals from best−fit line
of predicted RT vs Expt. scan number
Line: Z score of novel peptide
Z: 4.86
count
400
200
0
−10
−5
0
5
10
Z scores of residuals from best linear fit
